# Health effects of exposure to nano-TiO_2_: a meta-analysis of experimental studies

**DOI:** 10.1186/1556-276X-8-51

**Published:** 2013-01-25

**Authors:** Xuhong Chang, Yu Zhang, Meng Tang, Bei Wang

**Affiliations:** 1 Key Laboratory of Environmental Medicine and Engineering, Ministry of Education, School of Public Health, Southeast University, Nanjing 210009, China; 2 Department of Epidemiology and Health Statistics, School of Public Health, Southeast University, Nanjing 210009, China; 3 Jiangsu Key Laboratory for Biomaterials and Devices, Southeast University, Nanjing 210009, China

**Keywords:** Health effects, Nano-TiO_2_, Meta-analysis, Toxicity, Epidemiology

## Abstract

The paper is aimed to investigate the toxicity of nano-TiO_2_ and its potential harmful impact on human health using meta-analysis of *in vitro* and short-time animal studies. Data were retrieved according to included and excluded criteria from 1994 to 2011. The combined toxic effects of nano-TiO_2_ were calculated by the different endpoints by cell and animal models. From analysis of the experimental studies, more than 50% showed positive statistical significance except the apoptosis group, and the cytotoxicity was in a dose-dependent but was not clear in size-dependent manner. Nano-TiO_2_ was detained in several important organs including the liver, spleen, kidney, and brain after entering the blood through different exposure routes, but the coefficient of the target organs was altered slightly from animal models. It is possible that nano-TiO_2_ can induce cell damage related to exposure size and dose. Further studies will be needed to demonstrate that nanoparticles have toxic effects on human body, especially in epidemiological studies.

## Review

### Background

Nanotechnology refers to a new set of technologies that are used to develop nanoscale structures and devices (typically between 1 and 100 nm at least in one dimension) with unique or enhanced properties utilized in commercial applications [1]. Nanotechnology promises to contribute much to the betterment of humanity, but without appropriate assessment of the risks, the technology will not be developed safely and with public confidence [2]. Concern has been raised about the potential impact of nanomaterials exposure on human health [3,4]. A paper reported that a large number of workers are potentially exposed to nanoparticles and the number will be larger as nanotechnology develops and spreads in Italy. Knowledge of exposure assessment shows that it is very important to boost research in this field [5]. The market may now face a growing number of downstream users who are not informed about the type and content of NPs in the products they use. A 2009 survey indicates that 80% of the workers’ representatives and 71% of the employers’ representatives were not aware of the availability of nanomaterials and were ignorant as to whether they actually use nanomaterials at their workplace [6]. If an industrial material is identified as a harmful material, the use may be restricted and the exposure may be minimized by mandating protective clothing and respirators.

Titanium dioxide (TiO_2_) is a widely used industrial nanomaterial in things such as sunscreens, lacquers, and paints [7]. The risk assessment of Nano-TiO_2_ should be an integral part of modern society. So we consider the following questions from a public health perspective: what organs will detain nano-TiO_2_ by different exposed routes, what effects do nano-TiO_2_ cause in the body, and what is the biological mechanism driving TiO_2_ nanoparticles toxicity? Epidemiologic studies form an important link in understanding health outcomes associated with exposures to potentially hazardous materials [2]. Population-based studies about nano-TiO_2_ are few [8]; only a number of articles examining the health risk of exposure to nano-TiO_2_ have been published on the subject from animal and cell experiment, but no coherent images can be achieved. Thus, a special paper on the combined effects could increase the knowledge on the toxicity of nano-TiO_2_ by meta-analysis.

## Methods

### Search strategy and inclusion criteria

The primary interest of this study is human health effects exposed to nano-TiO_2_. Since there were no epidemiological studies on the subject, we have considered experimental studies employing human cells, animals, and animals cells as experimental units and exposing them to nano-TiO_2_. The study articles must have definite purpose, biological model, exposure time, exposure dose, nano-TiO_2_ diameter (less than or equal to 100 nm), type of endpoint measured, and main results. A comprehensive literature search of several databases (pubmed, web of science, CNKI, VIP, etc.) was conducted with combination of relevant keywords, such as nano, titanium dioxide, health effects, toxicity, mice, rat, experiment, human, stress, lactate dehydrogenase, and enzyme kinetics. Only articles published in English and Chinese were used. Abstracts and review articles were not included.

### Data extraction

From all the studies, we documented the following items for the description of evidence: (a) biological model: the category of studying cells or animals; the material character included physical and chemical properties especially diameter; (b) study design: exposure time, dose, and routes of nano-TiO_2_ into the biological systems; and (c) main results: the study endpoints from cell models (cytotoxicity, enzyme activities, genotoxicity, apoptosis, inflammation, etc.), and animal models (target organ, the change of Ti detain and different organ coefficients etc.). The data were extracted independently from each article by two members of the research, and the discrepancies in the information were resolved by consensus meetings.

### Meta-analysis methods

Because of the great variety of the cell types or animal species used and endpoints measured in different studies, calculation of a summary estimate of the effect size was not possible. A very simple approach based on the proportion of studies with positive findings from the same endpoints was used. The studies were classified as ‘positive study’ (exposure to nano-TiO_2_ group had statistical significance compared with the control group in one of the endpoints) and ‘negative study’ (no statistical significance). The analysis involved the percentage of positive studies for categories according to various experimental characteristics. It is important to note that a given study could be positive in one category, but negative in another category. A particular study could include both positive and negative findings, if more than one experiment was performed with varying cell lines, exposure schedules, etc., or if more than one biological endpoint was measured. Analyses were made to examine whether the percentage of positive studies was dependent on the following: biological agent used, type of endpoint measured, dose and time of exposing nano-TiO_2_, exposed route, and nano-TiO_2_ diameter.

## Results

### Identification of studies

**Figure 1:**
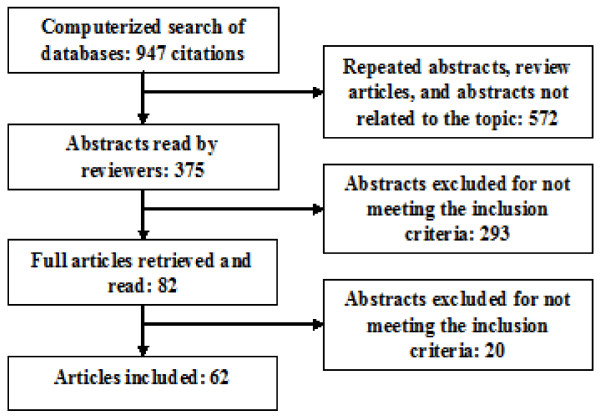
Article selection flow chart.

The electronic search resulted in 947 citations (Figure 1). 375 articles were selected after eliminating repeated abstracts, review articles, and non-related topic articles. After applying the inclusion criteria, 82 articles were selected, retrieved, and read. Finally, 62 articles were chosen for inclusion into the meta-analysis study.

### Description of the evidence

One study included both cell and animal models, and the description of evidence is documented in Table 1 (27 studies on cell models) and Table 2 (26 studies on mice and rats) for the studies investigating the behavior of different biological model when exposed to nano-TiO_2_.

**Table 1:** **Description of evidence for health effects of nano-TiO**_**2 **_**from cells models**

**Reference**	**Biological model**	**Diameter (nm)**	**Time (h)**	**Dose**	**Main results**
[9]	U937	100	24~48	0.005~4 mg/ml	Apoptotic and necrotic modifications
[10]	A549	63	4~18	80 μg/ml	DNA damage
[11]	A549/NCI-H1299	20	24	0.3~1,000 μg/ml	Aggregated
[12]	CHO/293 T	10~60	24	25~325 μg/ml	Genotoxicity
[13]	BEAS-2B	5	24	100 μg/ml	Increases cytokines IL-6 and IL-8
[14]	HDF/A549	3.2~10	48	0.3~3,000 μg/ml	Cytotoxicity and inflammation
[15]	U973	20	12~24	0.625~20 μg/ml	Transcriptional change of TIMP-1
[16]	BGC-823	20	24~72	100~800 mg/L	Cytotoxicity and inhibited growth
[17]	NIH3 T3/HFW	15	24~72	0.0005~50 μg/ml	Cytotoxicity and ROS
[18]	WIL2-NS	8.2	6~48	26~130 μg/ml	Cause genotoxicity and cytotoxicity
[19]	PC12 cells	21	6~48	1~100 μg/ml	ROS and apoptosis
[20]	lymphocytes	25	1~48	20~100 μg/ml	Induced genotoxicity
[21]	MC3T3-E1	5/32	24~72	5~500 μg/ml	Cytotoxicity and pro-inflammatory
[22]	Hela cells	80 × 10	12	0.1~1.6 mg/ml	Cytotoxicity and OS-mediated
[23]	THP-1 cells	10 to 40	24	0.1~1.6 mg/ml	Reactive oxygen
[24]	HDMEC	70	24~72	5~50 μg/ml	No cytotoxicity and inflammatory
[25]	CHL	21	24/72	0.025~1.00 mg/ml	Cytotoxicity
[26]	HLF	21/80	24/48	5~80 mg/L	Inhibit GJIC
[27]	A549	5 to 10	6	25~200 μg/ml	DNA damage
[28]	Red cells	15	3	1.25~20.0 g/L	MDA generations and hemolytic
[29]	A549	25	1~24	100 μg/ml	ROS and inhibit the growth
[30]	BGC-823	20	24	0.1~0.4 mg/ml	Increased ROS levels
[31]	HaCaT	20 to 35	4	10~300 μg/ml	Damaged structure and inhibited growth
[32]	A549	5	24~72	5~160 μg/ml	Induced ROS
[33]	L929	20 to 100	24~72	50~200 μg/ml	No cell proliferation and apoptosis
[34]	293 T and CHO	10	24	10~500 μg/ml	Induced cell apoptosis
[35]	HaCaT	4~60	24	10~200 mg/ml	Cytotoxicity and apoptosis

BEAS, Human bronchial epithelial cells; CHL, Classical Hodgkin lymphoma; HDMEC, Human dermal microvascular endothelial cells; GJIC, Gap junctional intercellular communication; HDL, human diploid fibroblast; HLF, Human lactoferrin; OS, Oxidative stress; NS, Nervous system; ROS, Reactive oxygen species.

**Table 2:** **Description of evidence for health effects of nano-TiO**_**2 **_**from mice and rats models**

**Reference**	**Exposed routes**	**Diameter (nm)**	**Dose**	**Time**	**Main results**
[36]	Digestive tract	25~155	5 g/kg	2 weeks	Transported to other tissues and organs
[7]	Respiratory tract	21	42 mg/m^3^	8 to 18 days	Lung inflammation and neurobehavioral toxicity
[37]	Respiratory tract	10/100	500 μg/mouse	30 days	Pathological lesions in the brain and neurotoxicity.
[38]	Intraperitoneal	5	5~150 mg/kg	14 days	Liver toxicity, inflammation, and apoptosis
[39]	Respiratory tract	25	1.25 mg	7 days	Lung toxicities and presence of aggregates or agglomerates
[40]	Skin	4/60	5% TiO_2_	60 days	Retained in the stratum corneum and the basal cells
[41]	Intraperitoneal	5	5~150 mg/kg	14 days	Liver DNA cleavage and hepatocyte apoptosis
[42]	Intraperitoneal	100	324~2592 mg/kg	7/14 days	The toxicity of the liver, kidney, lung, and spleen
[43]	Intraperitoneal	5	5~150 mg/kg	14 days	Caused serious damage to the liver and kidney
[44]	Respiratory tract	<10	5~500 μg	24 h	Induce lung inflammation
[45]	Respiratory tract	34.8	550 μg/m^3^	4 h	Do not induce lung inflammation
[46]	Digestive tract	20 to 30	5 g/kg	14 days	Liver and kidney toxicity
[47]	Intraperitoneal	30	200~500 mg/kg	17 days	Liver, kidney, and male productive toxicity
[25]	Intraperitoneal	21	300 mg/kg	18 h	Lung and liver damage
[48]	Intraperitoneal	30	300 mg/kg	18 h/10 days	No histopathological change in the tissue
[49]	Intraperitoneal	20~40	4.876~120.7 mg/kg	14 days	Liver damage
[50]	Respiratory tract	25	1~10 mg/kg	10 days	Lung damage
[51]	Intraperitoneal	30	200~500 mg/kg	17 days	Slight damages in the liver, kidney, and heart
[52]	Digestive tract	20 to 30	5 g/kg	14 days	Liver and kidney toxicity
[53]	Respiratory tract	10	1,500 mg/m3	7~28 days	Increased in pulmonary inflammation
[54]	Caudal vein	20 to 100	0.1~0.8 mg/ml	5 days	Induce DNA damage of the liver and kidney
[55]	Digestive tract	4	5 g/kg	14 days	No change in coefficients of the organs
[56]	Intraperitoneal	6.9	5~150 mg/kg	14 days	Induced kidney toxicity
[57]	Respiratory tract	15	1~10 mg/kg	7~days	Lung injury, changed the enzyme activities
[58]	Caudal vein	5	0.24 μg/mouse	1~48 h	Increase content of Ti in the liver, lung, and spleen
[59]	Respiratory tract	80	-	1 month	Distribution of Ti in the neural system
[60]	Respiratory tract	50	0.5~50 mg/kg	7 days	Induced oxidative stress in the liver and kidney
[61]	Respiratory tract	20~30	3.5~17.5 mg/kg	5 weeks	Lung damage, oxidative effects, inflammation
[62]	Intraperitoneal	62	1~15 mg/kg	21 days	Nephrotoxicity and tubular damages
[63]	Respiratory tract	5	0.8~20 mg/kg	7 days	Liver and lung damage
[64]	Respiratory tract	5~10	0.4~40 mg/kg	7 days	Changed enzyme activities
[65]	Respiratory tract	25.1	2~50 mg/m3	5 days	Enzyme activities and induced lung toxicity
[66]	Respiratory tract	28.4	5 mg/kg	1 weeks	Lung damage
[67]	Respiratory tract	5	0.8~20 mg/kg	7 days	Aggregate in the lung and kidney
[68]	Respiratory tract	5, 21, 50	0.5~50 mg/kg	7 days	Pulmonary toxicity
[69]	Respiratory tract	20 to 30	3.5~17.5 mg/kg	5 weeks	Immune system toxicity

### The toxicity of nano-TiO_2_ from vitro studies

The cultured cells exposed to toxic agents can respond with various mechanisms that differ in the level of cell damage. Nano-TiO_2_ has been studied mainly with established *in vitro* toxicity assays that analyze major cellular parameters such as cytotoxicity, enzyme activities, genotoxicity, and response to various stress factors. Although a variety of cell studies using nano-TiO_2_ has been published so far, different articles may have no coherent results. In this study, we calculated the percentage of positive studies with several of important endpoints. The overall percentage of positive studies differed very significantly (*p* < 0.01) from the expected value of positive studies if there is no true effect (less than 5% of studies are expected to show a *p* value less than 0.05 just by chance), suggesting that we can reject the null hypothesis. According to Tables 3, 4, 5, the total percentage of positive studies was lower for studies on inflammation (25%) than for studies on other endpoints, and the group of genotoxicity had a highest percent positive result that reached 100% but based on small numbers.

**Table 3:** Cytotoxicity and enzyme activities in different times and doses

**Study dose (mg/ml)**	**Cytotoxicity**^**a **^**(h)**						**Enzyme activities**^**a **^**(h)**			
	**≤12**	**≤24**	**≤48**	**≤72**	**Total**	**Percentage**^**b**^	0~6*	24*	**Total**	**Percentage**^**b**^
≤0.005	0/2	1/10	3/5	1/3	5/20	20	2/1	0/4	2/5	29
≤0.05	3/2	10/10	9/4	4/5	26/21	55	3/0	4/1	7/1	88
≤0.5	7/1	15/7	10/2	5/2	37/12	76	3/0	5/0	8/0	100
≤5	1/1	5/1	3/1	3/0	12/3	80	1/0	2/0	3/0	100
≤50	1/0/	1/0	0/0	0/0	2/0	100	1/0	1/0	2/0	100
Total	12/6	32/28	25/12	13/10	82/56	59	10/1	12/5	22/6	79
Percentage^b^	67	53	68	57	-	-	91	71	-	-

^a^Number of positive/negative studies.

^b^Percentage of positive studies.

#### Cytotoxicity

Different endpoints for cytotoxicity have been used in nanomaterials toxicity testing. Metabolic activity, for instance, has been widely determined using the colorimetric MTT assay based on the reduction of a yellow tetrazolium dye (MTT) to a purple formation in the cells bearing intact mitochondria. Cellular necrosis is another endpoint commonly used in cell viability studies. Upon necrosis, significant amounts of LDH is released from the cytosol and this LDH release can be easily detected using INT (a yellow tetrazolin salt) as a substrate since LDH catalyze its oxidation to a red formation [70]. Grouping of the cytotoxicity studies showed cytoxicity in a dose-dependent manner and an inconspicuous time-dependent relationship (Table 3). The percentage of positive studies was more than 50% at over 0.005 mg/ml and in all study times. Especially the group at 50 mg/ml there were two positive studies from the papers, but this is based on small numbers.

#### Enzyme activities

Evidence is accumulating that enzyme activities induced by nanomaterials is a key route by which these nanomaterials induce cell damage. Our combined results clearly showed that exposure to nano-TiO_2_ could induce the change of enzyme activities, and the percentage of the positive studies have been relatively high at all study times and more than 0.005 mg/kg concentration. Overall, this results are based on small numbers and further study needs to be done (Table 3).

#### Genotoxicity

Evidence of genotoxicity has been previously researched within a number of studies; micronuclei development is associated with nano-TiO_2_ exposure, which is indicative of chromosomal damage; DNA damage has also been observed in response to nano-TiO_2_ exposure. The classic comet assay based on gel electrophoresis and the detection of *in vitro* mammalian chromosomal aberrations are the most commonly used test systems to assess genotoxicity. A review describes knowledge about genotoxicity investigations on nanomaterials published in an openly available scientific literature from all biological models [71]. In the following discussion, we focus on the nano-TiO_2_ genotoxicity from the cell model with a dose and time relationships, and all studies are positive based on the results of a small number studies (Table 4).

**Table 4:** Genotoxicity and apoptosis in the different times and doses

**Study hour**	** Genotoxicity**^**a **^**(mg/ml)**		**Apoptosis**^**a **^**(mg/ml)**						
	**≤0.05**	**≤0.5**	**≤0.005**	**≤0.05**	**≤0.5**	**≤5**	**≤50**	**Total**	**Percentage**^**b**^
≤6r	2/0	2/0	0/0	1/	2/	0/0	0/0	3/0	100
≤24	3/0	5/0	1/0	2/3	4/3	1/0	1/0	9/6	60
≤48	4/0	4/0	1/0	1/3	2/2	1/0	0/0	5/5	50
Total	7/0	11/0	2/0	4/6	8/5	2/0	1/0	17/11	61
Percentage^b^	100	100	100	40	62	100	100	-	-

^a^Number of positive/negative studies.

^b^Percentage of positive studies.

#### Apoptosis

Li et al. [72] revealed that there was the dose-dependent effect of apoptosis in the N9 cells exposed to nano-TiO_2_ and the significant difference observed in 16 μg/ml TiO_2_ NPs-treated groups and this apoptosis might lead to the dysfunction of microregions. The study of Carmen et al. [10] reported that suspensions of TiO_2_ nanoparticles prepared in U937 cells culture medium at concentrations that covered a range (0.005 to 4 mg/kg) induced apoptosis in 24 and 48 h. In contrast, Han et al. [33] results showed that the cell apoptosis was not influenced by the presence of nano-TiO_2_ at 50 to 200 μg/ml for 24 to 72 h. Different studies have different results and in this report on apoptosis, tests from cell models were summarized and we calculated the combined effects of exposure to nano-TiO_2_. According to Table 4, there is a combined apoptosis effects at different times and dosages and it gave us a clue for apoptosis induced by exposure to nano-TiO_2_, although the number of studies was small.

#### Inflammation

To assess inflammation by nanomaterials immunotoxicity, the production of inflammatory markers such as the chemokines interleukin (IL)-8, IL-6, or TNF-α was usually measured in cell culture supernatants using enzyme-linked immunosorbant assay. In this study, we realized that the percentage of positive study is lower and no dose- and time-dependent relationships were found, and this may due to the small number of studies available. Future studies determining inflammatory combined effects of nano-TiO_2_ need go deep into (Table 5) these aspects.

**Table 5:** Inflammation and cytotoxicity in 24 h for the different doses

**Study dose (mg/ml)**	**Inflammation**^**a **^**(h)**				**Cytotoxicity at 24 h**^**a **^**(nm)**					
	**≤24**	**≤48**	**Total**	**Percentage**^**b**^	**<10**	**10 to 20**	**21 to 40**	**40 to 100**	**Total**	**Percentage**^**b**^
≤0.005	0/1	0/2	0/3	0	0/2	1/6	0/3	0/2	1/13	/7
≤0.05	0/1	0/2	0/3	0	0/3	7/3	4/2	0/2	11/10	52
≤0.5	1/1	1/1	2/2	50	2/2/	5/2	5/2	0/2	12/8	60
≤5	0/0	1/1	1/1	50	0/0	3/1	1/1	1/0	5/2	71
Total	1/3	2/6	3/9	-	2/7	16/12	10/8	1/6	29/33	47
Percentage^b^	25	25	25	-	22	57	56	14	-	-

^a^Number of positive/negative studies.

^b^Percentage of positive studies.

#### Size dependency

Particle dimension is recognized as being fundamental to their toxicity. This derives from the fact that NPs have been consistently demonstrated to be capable of eliciting more pronounced toxicity than their large (microparticulate) counterparts [73]. The size dependency of nano-TiO_2_ toxicity has been frequently demonstrated and appears to be applicable to a variety of nano-TiO_2_ forms from the cell model. In this study, we summarized the cytotoxicity of different nano-TiO_2_ dimension at 24 h, and we found that the percentage of positive studies is higher at the 10 to 40 nm than other groups (Table 5).

### The toxicity of nano-TiO_2_ from vivo

#### Contents of Ti and coefficients from different organs

After entering the blood by absorption or various exposed route, nano-TiO_2_ was distributed to the important organs throughout the body. Distribution usually occurs rapidly; the rate of distribution to organs or tissues is determined primarily by blood flow and the rate of diffusion out of the capillary bed into the cells of a particular organ or tissue. In general, the initial phase of distribution is dominated by blood flow, whereas the eventual distribution is determined largely by affinity. Understanding the distribution of nano-TiO_2_ in the organs was the premise of studying toxicity and this will provide direct evidence. We calculated the percentage of positive studies based on different organs and time (Table 6). Those results suggested that nano-TiO_2_ can be distributed in the important organs and it is possible to inducing body damage for biological systems. Grouping of the studies of the spleen and brain revealed that the percentage of positive studies was higher than others. The contents of Ti in the heart are lower, but this is based on small number of studies. In different study times, every organ has a relatively higher content of Ti and at 14 days it reaches at 81%. According to the results of Table 6, we further calculated the coefficients of different organs and it showed that although exposure to nano-TiO_2_ could increase deposition of Ti in different organs, the coefficients of organs were changed slightly (Table 6). We draw a conclusion that Ti detention may not cause the change of coefficient of the targeted organs.

**Table 6:** Contents of Ti and coefficients in the different organs

	**Study time (day)**	**Liver**^**a**^	**Spleen**^**a**^	**Kidney**^**a**^	**Lung**^**a**^	**Brain**^**a**^	**Heart**^**a**^	**Total**^**a**^	**Percentage**^**b**^
Contents of Ti	≤7	4/2	3/0	1/2	5/1	0/1	1/1	14/7	67
	≤14	5/1	5/0	4/1	4/1	3/0	1/2	22/5	81
	≤28	0/2	0/0	0/0	2/1	1/0	0/0	3/3	50
	Total	9/5	8/0	5/3	11/3	4/1	2/3	35	15
	Percentage^b^	64	100	63	79	80	40	70	-
Coefficient	≤7	0/1	0/0	0/1	4/0	0/0	0/0	4/2	67
	≤14	9/13	2/10	4/10	4/6	3/7	1/9	23/55	29
	≤28	0/2	0/2	0/2	1/3	0/0	0/2	1/11	8
	Total	9/16	2/12	4/13	9/9	3/7	1/11	28/68	-
	Percentage^b^	36	14	24	50	30	8	29	-

^a^Number of positive/negative studies.

^b^Percentage of positive studies.

#### The toxicity of nano-TiO_2_ from the study of different exposed routes

Because exposure to nanoparticles can occur through inhalation, skin contact, ingestion, and injection, studies with biological model are the best possible approximation to exposure of the respiratory tract, skin, gastrointestinal tract, intraperitoneal injection, or caudal vein to nanomaterials. Studies found that exposure to nano-TiO_2_ through different routes induced several damages to the important organs, and the percentage of the positive studies was calculated (Table 7). Results of the combined effects showed that the positive percentage is higher in the exposure to nano-TiO_2_ in various routes and the majority of the studies in the exposure to nano-TiO_2_ group had statistical significance compared with the control group.

**Table 7:** **Combined effects of nano-TiO**_**2 **_**on various organs**

**Exposed route**	**Liver**^**a**^	**Spleen**^**a**^	**Kidney**^**a**^	**Lung**^**a**^	**Brain**^**a**^	**Heart**^**a**^	**Total**^**a**^	**Percentage**^**b**^
Digestive tract	3/0	0/1	3/0	0/1	1/0	0/1	7/3	70
Respiratory tract	4/0	1/1	2/1	12/3	1/1	0/2	20/8	71
Intraperitoneal injection	7/2	1/1	5/1	2/2	1/0	2/1	18/7	72
Skin	1/0	1/0	1/0	1/0	0/1	0/1	4/2	67
Caudal vein	1/0	0/0	2/0	0/0	0/0	0/0	3/0	100
Total^a^	16/2	3/3	13/2	15/6	3/2	2/5	52/20	-
Percentage^b^	89	50	87	71	60	29	72	-

^a^Number of positive/negative studies.

^b^Percentage of positive studies.

### The toxicity of nano-TiO_2_ from the study of different main organs

#### Liver toxicity

The liver is the main organ where exogenous chemicals are metabolized and eventually excreted. As a consequence, the liver cells are exposed to significant concentrations of these chemicals, which can result in liver dysfunction, cell injury, and even organ failure. Eighteen studies found the toxicity of nano-TiO_2_ in the liver from mice or rats, *in vivo*. The findings from the studies [36,46,52] after oral exposure suggested that nano-TiO_2_ could induce the damage to the liver and pathologic examination showed that in the liver tissue, the hydropic degeneration of the hepatocyte around the central vein was found, with hepatocyte disorder, superficial staining of cytoplasm osteoporosis. Tang et al. [67] investigated the liver toxicity of nano-TiO_2_ subsequent to the intratracheal instillation and indicated slight liver injury and induced oxidative stress. But no coherent results emerged, and so liver toxicity of the combined effects was calculated when exposed to nano-TiO_2_. The percentage of the positive studies is 89%, and it is very possible that exposure to nano-TiO_2_ causes a liver toxicity (Table 7).

#### Spleen toxicity

Immunotoxicology can be most simply defined as the study of the adverse effects on the immune system resulting from occupational, inadvertent, or therapeutic exposure to drugs, environmental chemicals, and, in some instances, biological materials. Studies in animals and humans have indicated that the immune system comprises potential target organs and that damage to this system can be associated with morbidity and even mortality. In this study, the spleen was chosen for understanding immunotoxicology induced by nano-TiO_2_ and the contents of Ti in spleen had increased significantly compared with the control group, but in the positive studies, the number of spleen coefficients was lower than other groups by only 14%. In six studies, three results showed nano-TiO_2_-induced spleen toxicity by different exposure routes (Table 7).

#### Kidney toxicity

The functional integrity of the mammalian kidney is vital to the total body homeostasis, because the kidney plays a principal role in the excretion of metabolic wastes and in the regulation of extracellular fluid volume, electrolyte composition, and acid–base balance. In this paper, we found that there is an association between kidney toxicity and nano-TiO_2_ because the Ti content and positive percentages are higher than other groups and the coefficient of kidney is relatively lower according to Table 6. The results suggested that the kidney may be a main target organ of exposure to nano-TiO_2_ through different routes into the body.

#### Lung toxicity

Adverse health effects of air pollution have been recognized in epidemiological studies, and it was found that ultrafine particles have been linked with pulmonary toxicity [74]. Here we focus on the pulmonary toxicity of exposure to nano-TiO_2_. Published articles about lung toxicity were obtained, and the available evidence supports that the percentage of positive studies is higher than other groups: 79% studies from the content of Ti in lung (Table 6), 50% from coefficient of lung (Table 6), and 71% from the combining effects by different exposure routes (Table 7).

#### Brain toxicity

Metal oxides have been extensively studied, because of their toxic effects on humans and their utility in the study of the nervous system (NS). For a review dedicated entirely to the toxicity of metal oxides, the reader is referred to [4,70,73]. In the following discussion, we focus on the most important organ, the brain, in the nervous system for nano-TiO_2_ exposure. Overall, the number of brain toxicity paper was very limited regarding the exposed nano-TiO_2_ by various routes. Four studies suggested that the contents of Ti increased at different exposure time (Table 6) and the coefficient of brain changed slightly (Table 6). According to Table 7, the results illustrated that the percentage of positive studies reached in 80%, but this is only based on a small number of studies.

#### Heart toxicity

Cardiovascular toxicology is concerned with the adverse effects of extrinsic and intrinsic stresses on the heart and vascular system. A limited number of studies have been conducted to determine the impact of nano-TiO_2_ particles within *in vivo* models of heart toxicity. However, the findings suggest that nano-TiO_2_ through different exposure routes is deposited in the heart and contribute to inflammatory response and change in the enzyme activities which leads to heart toxicity. Grouping of the studies with heart toxicity revealed that the percentage of positive studies was lower than other groups about Ti content, coefficient, and combined effects by different routes (Tables 6 and 7).

## Conclusion and discussion

Evaluating the hazards associated with nano-TiO_2_ is vital for risk assessments. Numerous articles from experiments have been reported in the literature on the relationship between exposure to nano-TiO_2_ and health consequences, but no coherent results have emerged from different articles. To reveal possible consistent patterns, 62 papers were collected and the data was analyzed by systematic comparison of the study characteristics between positive and negative studies. This paper provides a few clues for the hypothesis that nano-TiO_2_ has an impact on health in humans.

Cultured cells exposed to nano-TiO_2_ can respond to various mechanisms that differ in the level of cell damage, and we accumulated 27 studies from cell models on the relationship between nano-TiO_2_ and biological system toxicity. Based on the different endpoints, we calculated the combined toxic effects of exposure to nano-TiO_2_. The results suggested that the percentage of positive studies is more than 50%, except in the apoptotic group. The cytotoxicity was dose-dependent but not clearly size-dependent. We summarized that the cytotoxicity of different nano-TiO_2_ dimensions at 24 h and the percentage of positive studies is higher at the 10 to 40 nm than other groups. It is possible that nano-TiO_2_ causes cell damage related to the size and dose in different endpoints. Exposure to toxins can occur through inhalation, skin contact, ingestion, and injection; and we found that different exposure routes can lead to the higher percentage of positive studies from *vivo* study. After entering the blood by absorption or various exposure routes, nano-TiO_2_ was detained in the several important organs such as the liver, spleen, kidney, and brain, but the coefficient of target organ was changed slightly. The liver and kidney have a high capacity for binding many chemicals. These two organs probably concentrate more toxicants than all the other organs combined, and in most cases, active transport or binding to tissue components are likely to be involved. In our study, we also found that the liver and kidney had a higher percentage of positive studies when exposed to nano-TiO_2_.

Standard problems related to meta-analytic approaches, including publication bias, variable quality, and unrecognized confounding, might have affected our results. We also recognize that our study has a possible bias. Firstly, the limitation of this meta-analysis stems from the languages chosen. Secondly, our conclusions could be biased due to the fact that positive results obtained from experiments with identical experimental design to those with negative results are not published finally. Another reason for bias in our study is the fact that the articles included in this meta-analysis were only from *in vitro* or animal experiment. Despite these limitations, to our knowledge, this meta-analysis represents the largest and most comprehensive effort to assess the safety of nano-TiO_2_.

At the nanometer scale, certain materials exhibited new properties that do not exhibit in macroscale. These new size-dependent properties of nanomaterials represent both the promise of nanotechnology and the concern about the potential adverse health effects on workers, consumers, and environment. Epidemiologic studies have the potential to be quite valuable in determining links between different types of occupational exposure to nanomaterials and the development of health problems. In addition, if properly designed, these studies could provide the ability to identify adverse health outcomes much earlier than if not conducted. However, the lessons learned from the studies of other particulates (e.g., asbestos and fine particulates in air) suggested that early attention to the health effects in the context of epidemiologic studies should be considered as soon as possible [8]. In order to take preventive measures, reduce and eliminate adverse effects on health, and provide a theoretical basis for the safety evaluation of nanomaterials, further research should consider epidemiological study to explore the association between nanomaterials and health effects.

## Competing interests

The authors declare that they have no competing interests.

## Authors’ contributions

All authors read and approved the final manuscript.
